# Associations between burnout symptoms and social behaviour: exploring the role of acute stress and vagal function

**DOI:** 10.1186/s12889-022-13333-3

**Published:** 2022-05-05

**Authors:** Magdalena K. Wekenborg, LaBarron K. Hill, Pia Grabbe, Julian F. Thayer, Clemens Kirschbaum, Susan Lindenlaub, Ralf Arne Wittling, Bernadette von Dawans

**Affiliations:** 1 Department of Psychology, Chair of Biopsychology, Dresden, TU Germany; 2 Else Kröner Fresenius Center for Digital Health, University Hospital and Medical Faculty Carl Gustav Carus, Dresden, TU Germany; 3 Department of Psychiatry and Behavioral Sciences, Duke University Medical Center, Durham, NC USA; 4 Department of General Internal Medicine and Psychosomatics, Center for Psychosocial Medicine, Heidelberg University Hospital, Heidelberg, Germany; 5 Department of Psychological Science, School of Social Ecology, University of California, Irvine, CA USA; 6 ZNF - Center for Neuroscience Research NPO, Trier, Germany; 7 Department of Biological and Clinical Psychology, University of Trier, Trier, Germany

**Keywords:** Burnout, Cynicism, Prosocial behaviour, Heart rate variability, Acute stress

## Abstract

**Background:**

The study aimed to investigate the link between burnout symptoms and prosocial behaviour, as well as the role of acute stress and vagally-mediated heart rate variability (vmHRV) on this association.

**Methods:**

Seventy men were randomly assigned to either the stress or the control condition of the Trier Social Stress Test for Groups (TSST-G). Prosocial behaviour was assessed via a social decision-making paradigm during the respective TSST-G condition.

**Results:**

Correlation analyses revealed negative correlations between prosocial behaviour and burnout symptoms. Acute stress was also associated with reduced prosocial behaviour, whereas no interaction effects with burnout symptoms could be revealed. Exploratory analyses showed that vmHRV was negatively correlated with burnout symptoms during the social decision-making paradigm but did not mediate the link between burnout and prosocial behaviour.

**Conclusion:**

In conclusion, we report first experimental evidence that burnout symptoms are negatively associated with prosocial behaviour. Further studies are needed to explore the causal relations.

## Background

Epidemiological research indicates a high prevalence of burnout symptoms especially but not exclusively among healthcare providers and related professions [1, 2]. This high prevalence is especially concerning given the numerous severe negative consequences of burnout symptoms on an organizational (e.g., absenteeism [3]), societal (e.g., immense economic costs [4]), and individual level (e.g., cardiovascular diseases [5]).

Deterioration of social interactions take a key role in modern conceptualizations of burnout symptoms: On the one hand, interpersonal stressors have been described as the primary cause of burnout emergence [6]. On the other hand, in addition to emotional exhaustion and reduced personal accomplishment, a cynical attitude towards co-workers and clients (i.e., cynicism) is a key burnout sub-dimension according to the 11^th^ Revision of the International Classification of Diseases (World Health Organization, 2018). More precisely, it has been proposed that the burnout sub-dimension cynicism develops as an attempt to distance oneself from experiencing exhaustion, by keeping an emotional distance to other’s problems and feelings [7].

Despite the central role of deteriorated social interactions within the theoretical burnout concept, surprisingly little research has explicitly examined potential negative associations between prosocial behaviour and burnout symptoms. Prosocial behaviour is thereby understood as the usage of one’s own resources for the gain of others [8]. There are indications of negative associations between burnout symptoms and the provision of social support [9–11], as well as empathic abilities and social skills [12–17]. Yet, other research failed to find significant associations between burnout symptoms and prosocial behaviour [18].

In summary, it must be said that the concept itself is of high complexity comprising several domains (like personal aspects, acute stress, chronic stress, social interaction, physiological adaptions etc.) as well as their constant interactions. Several reasons may account for these inconsistent findings. First, as argued by Dahlman, Jonsdottir [19], it seems important to differentiate between studies that pursue burnout as a psychological concept within occupational settings (i.e., burnout symptoms) and studies that examined patients receiving professional treatment (i.e., burnout syndrome): Notably, patients may differ from non-patients with respect to the severity of burnout symptoms. Furthermore, since they are commonly on sick leave it can be assumed that they are less exposed to daily stressors which suggests the presence of further outcome relevant differences between these groups [19]. Given the lack of universally accepted diagnostic criteria for burnout, it seems advisable to focus on employed individuals which varied based on their burnout symptoms rather than on inconsistently diagnosed patients. Second, most of the previous studies were conducted within specific occupational groups, limiting their external validity. Third, as none of the previous studies used experimental paradigms which enable direct and objective observation of interactive behaviour, well-known short comings of self-reported data (e.g., social desirability response bias) might have influenced the results.

Forth, none of the previous studies examined potentially modulating factors of the plausible association between burnout symptoms and prosocial behaviour. Acute stress has been shown to modify prosocial behaviour (for review, see [8, 20]). The direction of this modulation is, however, ambiguous, strongly depending on characteristics of the individual [8, 20]. The level of burnout symptoms might be an individual characteristic especially relevant for the direction of this modulation. We and others have shown that burnout symptoms are associated with dysregulations in the core stress systems of the human body (autonomic nervous system [ANS] and hypothalamus–pituitary–adrenal [HPA] axis) under acute stress [21, 22]. Whether these burnout-associated modulations in the core stress systems of the body also influence prosocial behaviour under acute stress remains an open question.

Elucidating the underlying physiological mechanisms linking burnout symptoms and prosocial behaviour is relevant both in understanding the aetiology of burnout, as well as the interplay of stress and social behaviour within a broader framework. The autonomic nervous system, and particularly, measures of vagally-mediated heart rate variability (vmHRV) may be a valid candidate. HRV describes the variability in the interval length between consecutive heartbeats [23]. Due to differences in chemical signalling, the vagus as the most important nerve of the parasympathetic nervous system, has a much faster influence on the heart rate (HR) than the sympathetic nervous system, making high-frequency changes in HRV (vmHRV) a relatively pure measure of vagal activity [24]. The two most prominent theories of cardiac vagal control, namely the *polyvagal theory* [25, 26] and the *model of neurovisceral integration* [27, 28] suggest, that measures of vmHRV index the capacity for effective emotion recognition and regulation, important perquisites of the ability to empathize and connect with others. Empirical studies have further revealed significant associations between prosocial behaviour and enhanced basal vagal tone [29–32], as well as task-related vmHRV as a state marker [33–35]. In addition, higher basal and task-related vmHRV were found to promote similar constructs including social connectedness and social sensitivity [36–38]. Lastly, we and others have consistently shown that burnout symptoms are associated with reduced vmHRV cross-sectionally [39, 40] and longitudinal [41] for overview see [42]. Thus, it is plausible that burnout-associated reductions in vmHRV reflect similar reductions in prosocial behaviour.

Given these considerations, the present study sought to determine whether burnout symptoms would be associated with observable modulations in prosocial behaviour in an age- and occupationally- heterogeneous, yet relatively small male sample. Secondarily, we investigated the potential modulatory role of acute stress and vagal function on the association between burnout and prosocial behaviour.

We used a validated paradigm from behavioural economics to investigate different prosocial behavioural facets as well as a non-social control condition [43]. We hypothesized 1) that burnout symptoms would be negatively related to prosocial behaviour, and 2) that an acute psychosocial stress provocation (i.e., the Trier Social Stress Test for Groups [TSST-G] [43]) would modulate this association. Lastly, we explored whether basal and/or vmHRV during social interaction would mediate the association between burnout symptoms and prosocial behaviour.

## Methods

### Participants

Participants for the present study were recruited either through their participation in the Dresden Burnout Study [44], or via public media. Due to influences of female menstrual cycle and oral contraceptives on the psychobiological stress response [45, 46] as well as the potential effects of gender in social interaction paradigms [47, 48] we choose an exclusively male sample without self-reported mental or physical illness.

Exclusion criteria were previous participation in a study involving any version of the Trier Social Stress Test (TSST [43, 49]), and current unemployment (to ensure a certain level of functioning).^1^

Participants who met the inclusion criteria were allowed to participate in full the study (i.e., target participants). Participants who did not meet the inclusion criteria were included as social interaction partners for the target participants (i.e., interaction partners). This second group did not complete the stress or control condition off the TSST-G or other measures and was involved only in the social decision-making paradigm. Target participants received an initial 15€; whereas interaction partners received 5€ for participation. Both, target participants and interaction partners could earn additional money in the social decision-making paradigm (mean of additional gain [SD]: 16.58 [7.44] €). All participants gave written informed consent. The study protocol was approved by the ethics committee of the TU Dresden and conducted in accordance with the Declaration of Helsinki.

### The social and non-social decision paradigms

Prosocial behaviour was assessed using a social decision making paradigm from behavioural economics already previously tested in men and women [50, 51], which is described in detail elsewhere [51]. In the present study, target participants interacted anonymously with randomly assigned real interaction partners who were not part of a TSST-G condition.

Shortly summarized, the social decision-making paradigm included decisions on trust and trustworthiness, on sharing (with four decisions each), as well as a non-social risk lottery.^2^ Each decision reflected a binary choice (e.g., trust vs. no trust). The social decision-making paradigm consisted of two sets, which were randomized, whereas each set comprised two decisions on trust game, two decisions on trustworthiness, two decisions on sharing, and four rounds of the non-social risk lottery.

Both, the trust game and trustworthiness game were sequential two-player games. The starting player decides whether to trust (gain depends on the decision of the other player) or not to trust (both players receive the same number of MU = monetary units). If the player with the first move choses trust, overall more MU could be scored, provided that the second player was trustworthy. Participants played four variants of the game as the starting player (trust) and four variants of this game as player with the second move (trustworthiness).

In the sharing game, players had to choose between keeping all MU for themselves or to share with an interaction partner, giving the interaction partner no opportunity to influence the outcome.

In the non-social risk game, the target participant played on his own. Players had to gamble, choosing either a high risk or a low risk option. The results were determined by virtual dice throwing: rolling a 1, 2, or 3 resulted in the higher outcome, whereas rolling a 4, 5, or 6 resulted in the lower outcome.

For the analyses, results of the respective games were summed-up in the following behavioural scores: (1) *trust*, (2) *trustworthiness*, (3) *sharing*, and a (4) *non-social risk score*. In addition, we calculated the (5) *prosocial index* (ranging from 0 to 12), which comprises decisions reflecting trust, trustworthiness and sharing (sum score; each ranging between 0 to 4). For all social behavioural scores (trust, trustworthiness, sharing), higher values indicate higher prosocial behaviour.

For the non-social risk game, 1 point was given for each decision in favour of the risky game, which resulted in a minimum of 0 and a maximum of 8 (*non-social risk score*), with higher scores indicating more non-social risk behaviour.

MU earned from all decisions were disbursed after the experiment according to the following exchange ratio: 100 MU = 0.95€, based on von Dawans, Ditzen [50]. The experiment was programmed and conducted with z-Tree software [52].

### Procedure

**Fig. 1:**
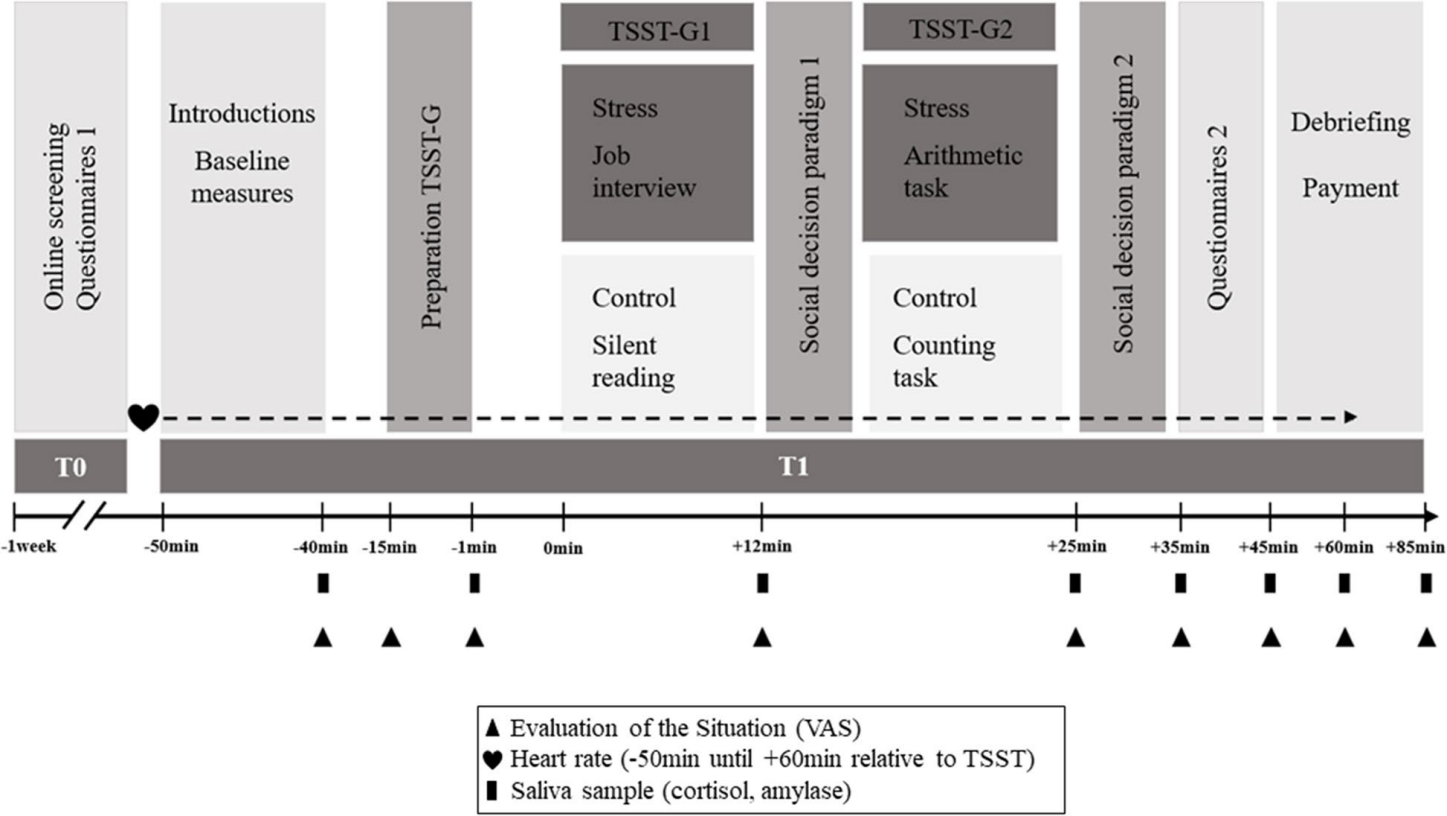
Flowchart of the experimental procedure for target participants. HR = heart rate; TSST-G = Trier Social Stress Test for Groups; VAS = visual analogue scale

The procedure for target participants followed the recommendations of von Dawans, Fischbacher [51] and is illustrated in Fig. 1. Participants were randomly assigned to either the stress (*n* = 35) or control condition (*n* = 35) of the TSST-G [43] and were invited to the lab in groups of four to six people. One week before the experiment, participants answered an online questionnaire on burnout symptomatology, sociodemographic characteristics and health-related factors (T0). One day before the experiment, participants were reminded via email to not take any medication and to abstain from alcohol, caffeine, sports, and smoking 24 h prior to the experiment. Moreover, they were told to eat standard meals and to abstain from food two hours prior to the experimental session. Experimental sessions started at 2 pm and lasted 2.5-h (T1). Upon arrival at the computer laboratory, participants were seated and were not allowed to communicate with each other. After reading and signing informed-consent forms, participants were introduced to the saliva-sampling method and were provided with a heart rate device (Polar RS800TM, Polar Electro, OY, Kempele, Finland) and baseline measures of salivary cortisol, HRV and psychometric variables were taken (additional measurements of acute stress were taken throughout the experiment as depicted in Fig. 1). After baseline measurement, participants received written instructions for the social decision making paradigm and were asked to complete control tasks to ensure the participants’ understanding of the paradigms. In the following, target participants were provided with the instructions for either the TSST-G stress or control condition and had 6 min to prepare for the mock job interview/group reading. Afterwards, they were guided to the test room. On their way, they were passed by the group of the interaction partners in order to guarantee the credibility of the existence of the human interaction partners. After arriving at the test room, target participants received a summary of the procedure.

As previously mentioned, target participants participated either in the stress condition of the TSST-G or a specific control condition, containing all factors of the stress condition except for the psychosocially stressful component, allowing the examination of the specificity of the stress effects [43]. In both conditions, participants stood next to one another, separated by partition walls, in front of a two-person committee that was evaluative only in the stress condition in order to induce socio-evaluative stress.

The exact sequence of tasks in the test room was: 12 min public-speaking task (stress condition TSST-G) or reading in a low voice (control condition TSST-G), followed by the first part of the social decision-making paradigm in a paper pencil version (5 min), then 8 min mental arithmetic task (stress condition TSST-G) or enumerating a series of numbers in a low voice (control condition TSST-G), followed by the second part of the social decision making paradigm (5 min; see also Fig. 1). After finishing the second part of the social decision making paradigm, target participants were guided back to the computer laboratory, where their previously made decisions had been matched with the interaction group’s decisions. At the end of the study participants were debriefed and received their compensation individually.

### Self-report measures

#### MBI-GS

Burnout symptoms were assessed with the German version (MBI-GS-D [53] of the Maslach Burnout Inventory-General-Survey (MBI-GS [54]), the most frequently used burnout measure in the field. The inventory comprises 16 items forming three sub-dimensions (emotional exhaustion, cynicism, reduced personal efficacy). All items are scored on a 7-point frequency-rating scale (0 = never; 6 = daily). The weighted MBI total score (0.4 × emotional exhaustion + 0.3 × cynicism + 0.3 × reduced personal accomplishment), introduced by Kalimo, Pahkin [55], and the three MBI sub-dimensions were considered as continuous variables and analysed separately. High scores on these scales indicate high burnout symptomatology.

#### PHQ-9

Depressive symptoms were measured with the German version (PHQ-D [56]) of the Patient Health Questionnaire (PHQ-9 [57]), which comprises the nine diagnostic criteria for depressive disorder of the Diagnostic and Statistical Manual of Mental Disorders [58]. The items are scored on a 4-point ranking scale (0 = not at all, 3 = nearly every day) and added up to a sum score. High scores on these scales indicate high depressive symptomatology.

### Assessment of biological measures

#### Autonomic measures

HR of target participants was recorded continuously via a chest belt throughout the whole experimental procedure (Polar RS800TM, Polar Electro, Oy, Kempele, Finland), and transferred to the Polar Precision Performance Software (Polar Electro OY, Kempele, Finnland). Researchers at the Center for Neuroscience Research (ZNF gGmbH, Trier, Germany) analysed the raw peak-to-peak intervals to calculate the HRV parameters according to the guidelines of the Task Force of the European Society of Cardiology and the North American Society of Pacing and Electrophysiology [23] and their specific implementation in the “NEUROCOR® presicionHRV-Algorithm Pat”. In the further process the HRV parameters of the intervals were only considered in the course of the evaluation if the artefact rate of the respective segment was below a given threshold in all valid neighbouring IBIs. Thus first, occurring artefacts were corrected by a smoothed spline interpolation and the artefact rate was calculated per interval. Second, in order to assess autonomic stress levels, mean HR (mHR) was calculated for each period of 90 s of continuous data collected throughout the experiment. Third, in order operationalize vagal function, the root mean square of successive differences of R-R-intervals (RMSSD) was calculated for 5 min of continuous data during each of the two sets of the social decision-making paradigm (vmHRV task). RMSSD was used as it has been shown to be less affected by breathing patterns and therefore more robust than high-frequency HRV [59, 60]. As previous research has been inconclusive regarding its influence on behavioural variables, basal as well as task-related vmHRV were considered within the present study. Basal vmHRV was operationalized as data collected during a 5 min seated resting condition before the introduction of the respective TSST-G condition. The two 5 min segments recorded during the two sets of the social decision making paradigm were averaged and are henceforth referenced as vmHRV task.^3^ As basal vmHRV and vmHRV task were not normally distributed, log transformation was applied to reduce skewness.

### Statistical analyses

As the behavioural scores (*trust, trustworthiness, sharing, non-social risk score*) were not normally distributed, all analyses involving these scores were conducted using non-parametric statistics.

In order to test our first hypothesis regarding associations between burnout symptoms and prosocial behaviour, partial correlation analyses between the four MBI scores, the behavioural scores, as well as the *prosocial index* were conducted, adjusting for age and depressive symptoms. To minimize multiple testing, only those MBI scores exhibiting a significant relation to the prosocial index^4^ were considered for the subsequent moderation and mediation analyses.

Next, we examined direct effects of acute stress on prosocial behaviour using a non-parametrical MANCOVA, with the behavioural scores and the *prosocial index* as the dependent variables, TSST-G condition (stress vs. control) as the independent variable and age as a covariate.

The second hypothesis regarding potential interaction effects between acute stress and burnout symptoms on the *prosocial index* were tested by conducting moderator analyses using the PROCESS macro [61], with the respective MBI score as the independent variable (X), the TSST-G condition (stress vs. control) as the moderator (M), and the prosocial index as the dependent variable (Y), adjusting for age and depressive symptoms.

Finally, in order to evaluate the role of vagal function, in a first step, we examined associations between the MBI scores, basal vmHRV and vmHRV task using partial correlation analyses, adjusting for age and depressive symptoms. To minimize multiple testing, only the vmHRV exhibiting a significant relation to any of the MBI scores was considered in further analysis. In a second step, the potential indirect effect of burnout symptoms on prosocial behaviour through vmHRV was evaluated, using the PROCESS macro [61]. Specifically, we estimated the conditional indirect effects of burnout symptoms (X) on social behaviour (Y) through vmHRV (M). A confidence interval including zero means that no evidence for mediation is given [61]. All statistical analyses were conducted using IBM SPSS Statistics v. 22 (SPSS Inc., Chicago, IL, USA), significance was set at *p* < 0.05, all tests were two-sided. Overlap between burnout and depressive symptoms remains a debated topic (for review see [62]), thus all analyses including burnout symptoms adjusted for depressive symptoms.^5^ As age has been shown to be associated with alterations in vmHRV [63] and burnout symptoms [64], it was considered as an additional covariate in these analyses,^6^ too.

## Results

### Descriptive statistics

The final sample (*N* = 70; mean [*SD*] age = 38.49 [11.39] years]) included 35 participants in the stress condition and 35 participants in the control condition of the TSST-G.^7^ The stress and the control condition of the TSST-G did not significantly differ in relevant demographic and health related characteristics (Table 1). The severity of the burnout symptoms of the participants varied between a 0.26 and 4.23 (mean [*SD*] MBI total score = 2.22 [0.97]) indicating a wide range of burnout symptoms, which was following the cut-off values proposed by Kalimo, Pahkin [55], subclinical on average.

**Table 1:** Demographic and health characteristics (*N* = 70)

Characteristics	Control (*N* = 35)	TSST-G (*N* = 35)	Group comparison
Demographics, *M* (*SD*)	40.34 (10.76)	36.63 (11.85)	*t*(68) = 1.37
Age, y			
BMI, kg/m^2^	24.64 (3.84)	24.63 (4.08)	*t*(68) = 0.02
Health-related factors, *n* (%)			
Smokers	7 (20.0)	5 (14.3)	*X*^2^(1) = 0.53
Daily alcohol consumption	9 (25.7)	6 (17.1)	*X*^2^(1) = 0.38
MBI total score, *M* (*SD*)	2.32 (1.00)	2.12 (0.96)	*t*(68) = 0.90
Emotional exhaustion	2.71 (1.23)	2.68 (1.47)	*t*(68) = 0.09
Cynicism	2.19 (1.26)	1.73 (1.13)	*t*(68) = 1.64
Reduced personal accomplishment	1.94 (1.15)	1.76 (1.07)	*t*(68) = 0.70
PHQ-9, *M* (*SD*)	7.09 (4.18)	7.11 (4.54)	*t*(68) = -0.28
Salivary cortisol (baseline)	4.89 (3.20)	6.22 (7.89)	*t*(68) = -0.48
Mean VAS (baseline)	2.09 (1.01)	2.54 (1.50)	*t*(68) = -1.47

*MBI total score* Maslach Burnout Inventory—General Survey total score, *mHR* mean heart rate, *PHQ-9* Patient Health Questionnaire, depression sum-score, *Mean VAS* mean value of six visual analogue scales. None of the group comparisons were significant

### TSST-manipulation check

We have previously demonstrated that the stress manipulation through the TSST-G was successful with respect to psychological (visual analogue scale) and biological (mean heart rate, salivary cortisol, vmHRV) markers of acute stress [21]. In contrast, the present paper focusses on burnout-associated changes in prosocial behaviour.

Within the scope of the first publication, we demonstrated that the stress manipulation through the TSST-G was successful with respect to psychological (visual analogue scale) and biological (mean heart rate, salivary cortisol, vmHRV) markers of acute stress [21].

### Associations between burnout symptoms and prosocial behaviour

Testing our first hypothesis regarding associations between burnout symptoms and behavioral scores using non-parametric partial correlation (adjusting for age and the PHQ-9) revealed significant negative associations between the MBI total score and the behavioral sub-score *sharing* (*r* = -0.30; *p* = 0.014). The cynicism sub-score was negatively associated with the behavioral sub-score *sharing* (*r* = -0.36; *p* = 0.003), as well as with the *prosocial index* (*r* = -0.26; *p* = 0.03) No further significant associations were observed between the additional MBI sub-scores and behavioural scores (see Table 2).

**Table 2:** Partial correlations between burnout symptoms, prosocial behaviour data, and vagally-mediated HRV at baseline and during the social decision making paradigm adjusted for age and depressive symptoms (*N* = 70)

	MBI total score		MBI EE		MBI CY		MBI PEr	
	*r*	*p*	*r*	*p*	*r*	*p*	*r*	*p*
MBI total score								
MBI EE	0.77**	< .001						
MBI CY	0.75**	< .001	0.44**	< .001				
MBI PEr	0.64**	< .001	0.22	0.07	0.34*	< 0.01		
Basal vmHRV	-0.14	0.25	-0.06	0.64	-0.18	0.14	-0.08	0.52
VmHRV task	-0.24	0.05	-0.09	0.49	-0.30*	0.01	-0.15	0.22
Prosocial index	-0.19	0.13	-0.06	0.62	-0.26*	0.03	-0.15	0.23
Trust	-0.02	0.90	0.03	0.80	-0.11	0.39	-0.02	0.85
Trustworthiness	-0.18	0.14	-0.11	0.40	-0.19	0.12	-0.12	0.34
Sharing	-0.30*	0.01	-0.16	0.19	-0.36*	< 0.01	-0.21	0.09
Non-social risk score	0.17	0.16	0.15	0.24	0.16	0.18	0.14	0.25

MBI total score = Maslach Burnout Inventory—General Survey total score; MBI EE = emotional exhaustion sub-dimension; MBI CY = Maslach Burnout Inventory- General Survey cynicism sub-dimension; MBI PEr = reduced professional efficacy sub-dimension; Basal vmHRV = root mean square of successive difference between heart beats at seated baseline; vmHRV task = root mean square of successive difference between heart beats during the social decision making paradigm. * for *p* < .05; ** for *p* < .001

### Effects of acute stress on prosocial behaviour and its interaction with burnout symptoms

The non-parametrical MANCOVA revealed reduced prosocial behaviour following acute stress, compared to the control condition with respect to *trust* (*F*(1,67) = 6.92, *p* = 0.01; η^2^ = 0.09) and the *prosocial index* (*F*(1,67) = 6.05, *p* = 0.02; η^2^ = 0.08). No significant associations were found with *trustworthiness* (*F*(1,67) = 2.63, *p* = 0.11), *sharing* (*F*(1,67) = 1.22, *p* = 0.27) and the *non-social risk score* (*F*(1,67) = 0.43, *p* = 0.52).

Next, a moderation analysis was conducted to test our second hypothesis of potential interaction effects of burnout symptoms and acute stress on prosocial behaviour. Only cynicism was considered, as previous analyses revealed that only this burnout sub-dimension depicted significant associations with the *prosocial index*. As depicted in Table 3, the moderation analysis did not provide confirmation of an interaction effect of the TSST-G condition on the association between cynicism and the *prosocial index* (Model *R*^2^ = 0.16, *p* < 0.05/Δ*R*^2^ due to interaction = 0.002, *p* = 0.73).

**Table 3:** Moderation model of cynicism and acute stress predicting the prosocial index (*N* = 70)

	Estimates	*SE*	95% CI lower bound	95% CI upper bound	*p*
Age	-0.03	0.04	-0.12	0.05	.42
MBI CY	-1.24	0.57	-2.39	-0.09	.04*
Acute stress	-3.14	1.75	-6.63	0.35	.08
MBI CY × acute stress	0.27	0.77	-1.27	1.81	.73

*MBI CY* Maslach Burnout Inventory- General Survey cynicism sub-score, *CI *confidence interval. * for *p* < .05; ** for *p* < .001

### Exploratory analyses on relations with vagal function

**Fig. 2:**
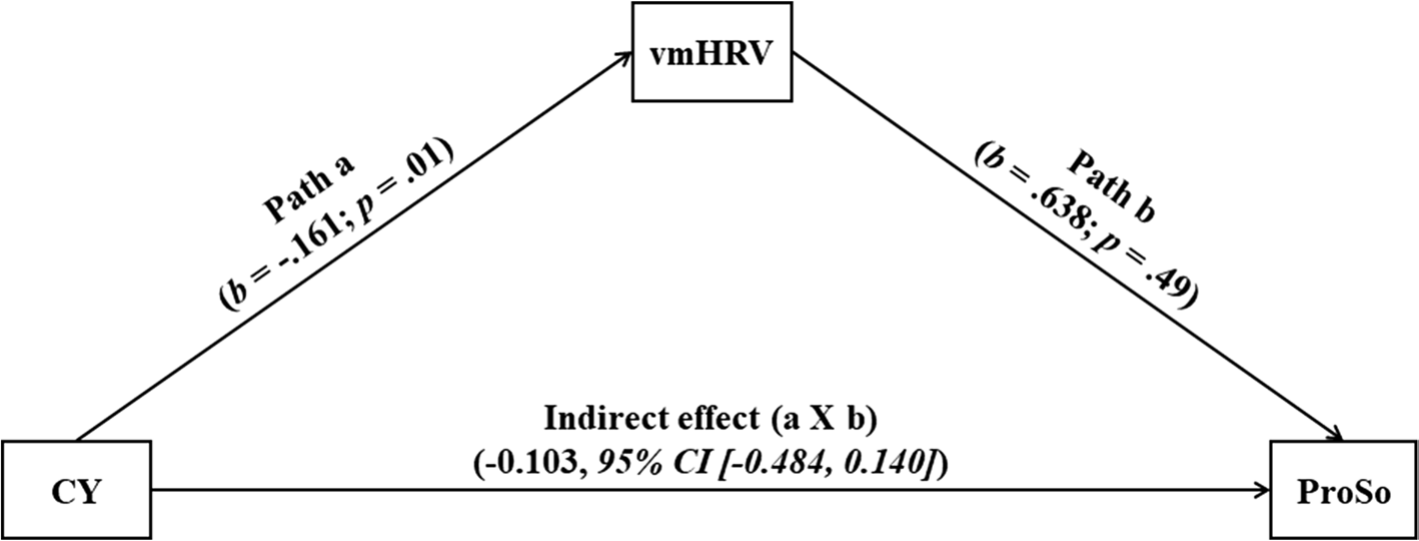
Mediation model of the association between burnout symptoms and the prosocial index through vmHRV during social interaction adjusted for age and depressive symptoms. CY = Maslach Burnout Inventory, cynicism sub-dimension; ProSo = prosocial index; vmHRV = vagally-mediated heart rate variability during social decision paradigm

Exploratory analyses were conducted to examine if burnout symptom-related changes in prosocial behaviour are linked to modulations in vmHRV. Partial correlational analyses revealed that cynicism was significantly negative associated with vmHRV during the social decision making paradigm (*r* = -0.30; *p* = 0.01), whereas no significant associations were observed between baseline vmHRV and any of the MBI sub-scores (see Table 2). As depicted in Fig. 2, the relationship between cynicism and the *prosocial index* was not mediated by vmHRV during the social decision-making paradigm.

## Discussion

The present study experimentally examined burnout-associated modulations in prosocial behaviour in an age and occupationally heterogeneous sample. Beyond this, we explored potentially modulating effects of acute stress, as well as underlying physiological mechanisms of this association. We found that burnout symptoms were negatively associated with *sharing* and a sum-score of the three behavioural scores *sharing*, *trust*, and *trustworthiness* (*prosocial index*) during a social decision-making paradigm. In addition, even though acute stress was associated with reduced prosocial behaviour, there was no additional interaction effect with burnout symptoms. Explorative analyses revealed that burnout-associated reductions in vmHRV paralleled burnout-associated reductions in prosocial behaviour. However, we did not find direct mediation effects of vmHRV on the association between burnout symptoms and prosocial behaviour.

With respect to our first hypothesis, significant negative correlations were found with respect to *sharing* and the *prosocial index* with the burnout sub-dimension cynicism. In addition, *sharing* was negatively associated with the MBI total score, indicating that higher burnout symptoms were associated with a reduced tendency for prosocial behaviour.

Importantly, there was no significant relation between any of the four MBI scores and non-social risk behaviour, suggesting that the effects are specific to social interactions. To the author’s knowledge our study is the first to indicate burnout-associated reductions in directly observable prosocial behaviour within a laboratory setting using a standardized social decision making paradigm. These findings are in line with previous questionnaire-based research revealing associations between burnout symptoms and reduced prosocial behaviour [18], enhanced hostile and impatient behaviour [65, 66], less prosocial organizational behaviour [67], more social conflicts [68, 69], and lower emotional intelligence [12, 14]. The finding of cynicism being the only burnout sub-dimension which showed significant negative associations with prosocial behaviour is in line with previous findings [70] and supports prior assertions that cynicisms reflects the interpersonal sub-score of burnout [71].

Our findings are highly relevant, given the well-known clinical consequences of reduced prosocial behaviour. For instance, from a stress-buffering perspective [72], positive social interactions have been repeatedly shown to reduce acute stress load [73] and to prevent mental diseases [74]. Burnout-associated reductions in prosocial behaviour may signal the beginning of a vicious circle of increasing burnout symptoms progressively reducing potentially stress buffering positive social interactions.

With respect to possible explanations for burnout-associated reductions in prosocial behaviour, by drawing on the conservation of resources theory (COR [75]) it has been proposed that burnout symptoms reflect a drain of resources and that the expression of negative social behaviour serves as a coping strategy in order to deal with these reduced cognitive and emotional resources [65, 76]. Following a similar line of reasoning, it has been proposed that individuals with higher burnout symptoms lack the resources to regulate self-centred motives in order to engage themselves in prosocial interactions with others [77, 78]. Our finding that emotional exhaustion was not significantly associated with reduced prosocial behaviour contradicts these theoretically derived assumptions in a way. However, for an actual verification of these theories, longitudinal studies are indispensable.

In addition, we were interested in potentially modulating effects of acute stress on the association between burnout symptoms and prosocial behaviour. Previous research showed that, depending on so far insufficiently explored individual characteristics, acute stress can modulate prosocial behaviour (for review see [8]). As burnout symptoms have been associated with modulations in acute stress reactivity [21, 22], it appears likely that burnout symptoms are one of these individual characteristics which influence the effects of acute stress on prosocial behaviour.

However, in contrast to our second hypothesis, we did not find any interaction effects between stress condition and burnout symptoms on prosocial behaviour. Interestingly, acute stress itself had a main effect on prosocial behaviour, in that subjects in the stress condition generally showed less prosocial behaviour compared to individuals in the control condition of the TSST-G. This finding is in line with a recently published review of 19 studies that induced acute stress in a laboratory setting and measured prosocial behaviour [8]. More precisely, Faber [8] reported that seven studies found a reduction of prosocial behaviour under acute stress, whereas only four studies found increases, three did not find any difference and five studies showed mixed results depending on moderating factors. The authors attribute this heterogeneity in study results to a complex interaction between acute stress, characteristics of the individuals, as well as the specific situation, which causes the individual to focus either on individual resources (gaining money through reduced prosocial behaviour; COR [79]) or social resources (gaining affiliation to social group through enhanced prosocial behaviour [8, 80]). One important difference between our and previous studies which found enhanced prosocial behaviour under stress [50, 51, 81] might be differences in the similarity with the social interaction partners. Previous research indicates more prosocial behaviour towards close members of the in-group but not to out-group others especially under acute stress [82]. In contrast to the age and occupationally heterogeneous sample of the present study, previous studies relied almost exclusively on rather homogenous samples of university students. Future studies are needed which examine the effect of conformity with regard to central demographic characteristic between interaction partners on prosocial behaviour under acute stress.

Finally, the finding of negative main effects of burnout symptoms and acute stress on prosocial behaviour with a simultaneous absence of a significant interaction between the two might suggest that burnout symptoms and acute stress contribute independently to prosocial behaviour.

As a more exploratory research question, based on the fact that vmHRV has been linked to both, enhanced prosocial behaviour [33], and reduced burnout symptoms [40, 41], this study investigated whether autonomic balance, as indexed by vmHRV during the social interaction paradigm mediated the link between burnout-related cynicism and prosocial behaviour. And in fact, we did find that reduced task-related vmHRV was associated with greater cynicism. However, there was no evidence for task-related vmHRV as a mediator between burnout-related cynicism and prosocial behaviour. In sum, the present finding of associations between reduced vmHRV and the interpersonal sub-dimension of burnout (i.e., cynicism) supports the important role of autonomic imbalance for social functioning. However, the fact that vmHRV was not directly linked to social behaviour indicates that the role of vagal function for observable social behaviour might be selective for specific kinds of prosocial social behaviour. Nevertheless, future studies investigating the effects of vagal-excitatory interventions on social behaviour in burnout (e.g., physical activity; breathing techniques, vagus nerve massage, and nicotine-free programs) may further elucidate this possible interaction.

There are also some limitations to be considered. First, in order to minimize potential confounders, the study included only employed individuals, which led to a restriction in severity of burnout symptoms. Second, considering that the development of a cynical attitude has been associated with male gender-role socialization [83], and that a meta-analysis has also revealed slightly higher cynicism scores in males [84], the inclusion of males only, taken together with the relatively small sample size, limit the generalizability of the results. Third, considering the cross-sectional study design, no assertions about the causal relationship between burnout symptoms and changes in social behaviour can be made. Forth, even though the central limit theorem suggests a normal distribution of a sample variable with sample sizes > 30 [85], results from parametric statistical test including the *prosocial index* (i.e., moderation and mediation analyses) should be interpreted with caution.

In sum, our study is the first to indicate negative associations between burnout symptoms and prosocial behaviour using a standardized social decision paradigm in a laboratory setting. In addition, we could show that the effects of acute stress on prosocial behaviour mirrored the results observed for burnout symptoms. The absence of interaction effects indicates a rather parallel influences of acute stress and chronic stress sequels (i.e., burnout symptoms) on prosocial behaviour. Finally, our finding of burnout-symptoms-associated reductions in vmHRV during the social decision making paradigm with a simultaneous absence of significant mediating effects of autonomic imbalance on the relation between burnout symptoms and prosocial behaviour, indicates that autonomic imbalance might play an important, yet more indirect role in burnout-associated modulations in prosocial behaviour.

Due to our small sample size, our findings should be interpreted with caution and should rather be seen as a starting point for longitudinal studies examining possible causal relationships between burnout symptoms and changes in social behaviour as well as their physiological underpinnings.

Moreover, future studies need to further disentangle the various interconnections of the highly complex construct of burnout. Besides longitudinal designs, and bigger sample sizes, future studies need to try to disentangle the role of various aspects like personal characteristics, chronic or acute stress and their interrelations. This may also lead to subgroups and a dimensional rather than a categorical approach to diagnostics in subclinical and clinical populations. It would be necessary to use multi-method and multi-center approaches, including psychological as well as physiological variables and investigate social interactive behaviours with high external validity in real person interactions. This may include open science practices in sharing designs and procedures as well as datasets. In addition, such approaches with the respective high sample sizes would also allow for including men, women and diverse participants over different age groups what would in turn allow for generalizability. Results of such studies would allow for better interpretation and conclusions.

Based on these studies, new therapy approaches may be developed focusing on the enhancement of positive social interactions as a powerful protective factor, which potentially compromised along the pathogenesis and course of burnout symptomatology.

## Data Availability

The datasets generated and/or analysed during the current study are not publicly available due to agreements among the authors but are available from the corresponding author on reasonable request.

## Abbreviations

ANS: Autonomic Nervous System
CY: Maslach Burnout Inventory, cynicism sub-dimension
HPA: Hypothalamus-Pituitary-Adrenal
HR: Heart rate
MBI-GS: Maslach Burnout Inventory-General-Survey
MBI-GS-D: German version of the Maslach Burnout Inventory-General-Survey
mHR: Mean heart rate
MU: Monetary units
TSST: Trier Social Stress Test
TSST-G: Trier Social Stress Test for Groups
vmHRV: Vagally-mediated heart rate variability
PHQ-9: Patient Health Questionnaire 9
PHQ-D: German version of the Patient Health Questionnaire 9
VAS: Visual analogue scale

## Footnotes

1 We are aware of the fact that the latter criterion limit the range of burnout symptom severity of participants included in the study, as very high levels of burnout symptoms are associated with an inability to work. However, as mentioned before, it has been suggested that studies should choose whether they examine burnout symptoms in a more or less healthy sample or in patients which have been seeking medical as results are not comparable due to difference in exposer to stressor resulting from sick leave/hospitalization. As no empirically validated burnout diagnoses has been established yet, we decided to focus on the former.

2 Ensures that possible changes in behaviour are specific to prosocial behaviour and cannot merely be explained by changes in risk behaviour.

3 In two cases HR was only recorded during one set of the social decision making paradigm due to technical problems. For these cases the missing values were replaced by the mean values of the respective other set of social decision making paradigm. Including them in analyses did not change the results.

4 We focussed on the prosocial index as in contrast to the behaviour sub-scores as, following the central limit theorem, the distribution of a sample variable approximates a normal distribution.

5 PHQ-9 was significantly associated with all MBI scores (MBI-total: *r* = .66, *p* < .01; cynicism: *r* = .53, *p* < .01; emotional exhaustion: *r* = .67, *p* < .01; reduced personal accomplishment: *r* = .53, *p* < .01).

6 All statistical analyses were conducted without elimination of outliers (values ± 2 *SD* of group means), as reported findings were the same regardless of whether outliers were excluded or not.

7 One participant was excluded from analyses because he was erroneously included, although unemployed.
